# Synergistic Antibacterial Effect of Ethyl Acetate Fraction of *Vernonia amygdalina* Delile Leaves with Tetracycline against Clinical Isolate Methicillin-Resistant *Staphylococcus aureus* (MRSA) and *Pseudomonas aeruginosa*

**DOI:** 10.1155/2023/2259534

**Published:** 2023-02-20

**Authors:** Denny Satria, Urip Harahap, Aminah Dalimunthe, Abdi Wira Septama, Triani Hertiani, Nasri Nasri

**Affiliations:** ^**1**^ Department of Pharmaceutical Biology, Faculty of Pharmacy, Universitas Sumatera Utara, Jl Tri Dharma No. 5 Kampus USU, Medan 20155, Sumatera Utara, Indonesia; ^2^Department of Pharmacology, Faculty of Pharmacy, Universitas Sumatera Utara, Jl Tri Dharma No. 5 Kampus USU, Medan 20155, Sumatera Utara, Indonesia; ^3^Research Center for Pharmaceutical Ingredient and Traditional Medicine, National Research and Innovation Agency (BRIN), Kawasan PUSPITEK Serpong, Tangerang Selatan, Banten 15314, Indonesia; ^4^Department of Pharmaceutical Biology, Faculty of Pharmacy, Universitas Gadjah Mada, Jl. Sekip Utara, Yogyakarta 55281, Indonesia; ^5^Department of Pharmacy, Sekolah Tinggi Ilmu Kesehatan (STIKes) Senior Medan, Jl. Jamin Ginting KM. 8,5 No. 13, Mangga, Medan Tuntungan 20141, Sumatera Utara, Indonesia

## Abstract

Multidrug-resistant bacteria have raised global concern about the inability to fight deadly infectious diseases. Methicillin-resistant* Staphylococcus aureus* (MRSA) and *Pseudomonas aeruginosa* are the most common resistant bacteria that are causing hospital infections. The present study was undertaken to investigate the synergistic antibacterial effect of the ethyl acetate fraction of *Vernonia amygdalina* Delile leaves (EAFVA) with tetracycline against the clinical isolates MRSA and *P. aeruginosa*. Microdilution was used to establish the minimum inhibitory concentration (MIC). A checkerboard assay was conducted for the interaction effect. Bacteriolysis, staphyloxanthin, and a swarming motility assay were also investigated. EAFVA exhibited antibacterial activity against MRSA and *P. aeruginosa* with a MIC value of 125 *μ*g/mL. Tetracycline showed antibacterial activity against MRSA and *P. aeruginosa* with MIC values of 15.62 and 31.25 *μ*g/mL, respectively. The interaction between EAFVA and tetracycline showed a synergistic effect against MRSA and *P. aeruginosa* with a Fractional Inhibitory Concentration Index (FICI) of 0.375 and 0.31, respectively. The combination of EAFVA and tetracycline induced the alteration of MRSA and *P. aeruginosa*, leading to cell death. Moreover, EAFVA also inhibited the quorum sensing system in MRSA and *P. aeruginosa.* The results revealed that EAFVA enhanced the antibacterial activity of tetracycline against MRSA and *P. aeruginosa*. This extract also regulated the quorum sensing system in the tested bacteria.

## 1. Introduction

The problem of antibiotic resistance is prominent worldwide. Multidrug-resistant bacteria make fatal infectious diseases untreatable, causing global concern [1, 2]. Methicillin-resistant *Staphylococcus aureus* (MRSA) is currently the most common resistant bacterium, especially in hospitals [3]. MRSA resistance to various antibiotics, including *β*-lactams, is caused by the acquired mecA gene, which overexpresses efflux pump and produces a *β*-lactamase enzyme [4]. In the case of Gram-negative bacteria, *Pseudomonas aeruginosa* has become a great concern as fatal bacteria resistance, due to changes in its target enzymes [5].

Several steps have been put in place to combat bacterial resistance. Thus, new antibacterial agents with unique targets and mechanisms of action are urgently needed. It is expensive and time-consuming to test new molecules in humans to make sure they are safe and effective in treating disease without fostering resistance [6]. Combining typical antibiotics with agents that enhance antimicrobial properties has been suggested as a possible solution to these problems [7]. On the other hand, it may be difficult to discover new antibacterial agents, and when the chemical is employed in clinical settings, new resistance mechanisms will develop. Combining two or more antimicrobials to increase their efficiency against resistant infections is a unique technique for combating resistance.

Numerous plant-based chemical compounds have been identified as a significant source of novel antibacterial. Several studies have identified chemical components, including flavonoids, fatty acids, sesquiterpene lactones, and steroidal saponins [8]. In addition, plant-based compounds also possess various biological activities for pharmaceutical action, such as anti-inflammation, antimalaria, antitumor, antiobesity, and antioxidant [9–12].

The *Vernonia amygdalina* Delile species is a member of the Asteraceae family [13] and is native to West Africa. Nonetheless, *V. amygdalina* leaf extract exhibited several pharmacological effects, including antibacterial activity [14]. However, this plant extract's effect on tetracycline's antibacterial activity against MRSA and *P. aeruginosa* is unknown. Thus, the present study aimed to examine the synergistic effect of the ethyl acetate fraction of *V. amygdalina* (EAFVA) extract and tetracycline on selected clinical isolates. In addition, the combination's effect on membrane cells and the virulence factor was studied [15].

## 2. Materials and Methods

### 2.1. Chemicals and Media

Tetracycline was obtained from Sigma-Aldrich, United Kingdom, phosphate buffered saline (PBS), anhydrous sodium sulfate, and crystal violet were obtained from Sigma-Aldrich, United Kingdom, brain heart infusion was obtained from BHI, and agar was obtained from Becton, Dickinson & Company, Franklin Lakes, New Jersey, USA.

### 2.2. Bacterial Strains

The clinical isolates obtained from the MERO Foundation (Marine Education and Research Organization Foundation in Bali, Indonesia) were MRSA and *P. aeruginosa*.

### 2.3. Preparation of Ethyl Acetate Fraction of *Vernonia Amygdalina* Delile Leaves

500 grams of *V. amygdalina* Delile were taken. The reflux technique extracted air-dried and powdered leaves with 1:10 absolute ethanol for five hours. We collected the filtrate, evaporated it at a lower pressure to produce a viscous extract, and finally dried it in a water bath [16, 17]. Using the process of liquid-liquid extraction, ethanol extract was fractionated with ethyl acetate [18, 19].

### 2.4. Determination of Minimum Inhibitory Concentration

In order to determine the MIC, a microdilution test was performed. The bacterial suspension was present and subsequently added to a 96-well microplate containing a two-fold dilution of *V. amygdalina* Delile. The bacterium suspension was prepared and adjusted to 0.5 Mc. Farland was equivalent to turbidity 1 × 10^8^ CFU/mL and then diluted using saline solution to generate 1 × 10^6^ CFU/mL [20]. Ethyl acetate fraction (EAFVA) was added to the leaves and cultured for 24 hours at 37°C [21]. It was thought that the MIC was the lowest concentration at which growth could be stopped [22].

### 2.5. Checkerboard Assay

On the *x*-axis of the 96-well plate, twice as much brain heart infusion (BHI) was added to the EAFVA. On the *y*-axis, an antibiotic dilution was created that was twice as potent as the previous one. Afterward, each well was given a suspension of bacteria at a concentration of approximately 1 × 10^6^ CFU/mL, and the mixture was incubated at 37°C for a full day. Calculating the Fractional Inhibitory Concentration Index (FICI) allowed us to investigate the interaction between EAFVA and antibiotics. The formula for calculating the FICI is as follows:

(1) $$\begin{array}{c} FICI=\frac{MIC\;of\;EAFVA\;or\;antibiotics\;in\;combination}{MIC\;of\;EAFVA\;or\;antibiotics\;alone}\text{.} \end{array}$$

This study was conducted to find out how EAFVA and antibiotics affected the tested bacteria [23]. The FICI calculation yields synergy when the FICI is less than 0.5. On the other hand, it was additive if the FICI value was in the range of 0.5 to 1, indifferent if the FICI value was 1 to 4, or antagonistic if the FICI value was greater than 4 [24].

### 2.6. Loss of 260 nm Absorbing Material

With a modest modification, the release of the UV-absorbing materials was accomplished, as previously reported. The overnight culture of the studied bacteria was washed and resuspended in the saline solution. To reach a final count of approximately 5 × 10^7^ CFU/mL, various concentrations of the substance were administered to the cell at 125 *μ*g/mL of EAFVA, 15.62 *μ*g/mL of Tetracycline, and the combination (15.62 *μ*g/mL EAFVA + 3.9 *μ*g/mL tetracycline).

Control cells were untreated. Each sample was incubated at 37°C for 24 hours, diluted with saline (1: 100), and filtered through a 0.2 *μ*m pore-size membrane. Spectrophotometer UV-VIS measured 260 nm optical density. This exam was taken three times [25].

### 2.7. Bacteriolysis Activity

The cultured bacterial suspensions were one night old on BHI media. Detection of bacteriolytic activity by this approach had been reported. 125 *μ*g/mL EAFVA, 31.25 *μ*g/mL tetracycline, and the combination (7.8 *μ*g/mL EAFVA + 7.8 *μ*g/mL tetracycline) were added to the cells. The final cell concentration was 5 × 10^7^ CFU/mL, whereas the control cells were not treated with the test concentration as a negative control. Then, they incubated and measured absorbance at OD 620 nm, which showed a decrease in absorbance. The calculated yield value was the percentage of absorbance versus the OD of 620 nm at 24 hours. The test was carried out three times with no errors [25].

### 2.8. Staphyloxanthin Assay

The ability of EAFVA to stop the production of the golden-yellow pigment and staphyloxanthin was being studied. Bacterial cultures were rejuvenated overnight in a lactose broth (LB) medium. Then the bacterial suspension was diluted in a ratio of 1: 100 in new LB media, which already contained EAFVA and MRSA. It was incubated at 37°C for 24 hours. EAFVA and negative control without treatment were centrifuged for 15 minutes at a speed of 10.000 rpm [26].

### 2.9. Swarming Motility Assay

Agar plates containing 24 mM CaCl_2_ were prepared for swarming agar plates (M8) containing 0.1 percent casamino acid, 0.5 percent glucose, and EAFVA. 20 mL of ready-made M8 media was put into each Petri dish. Let stand at room temperature until the consistency of the media solidified before using. 1 mL of the bacterial suspension cultured overnight was taken and then spun down at 6000 rpm for 3 minutes. Again, the cell pellet was spun down while suspended in 1 mL PBS, discarding the supernatant. This washing technique was carried out twice. Furthermore, bacteria were placed in the center of M8 medium and incubated for 10 minutes at room temperature [27].

### 2.10. Phytochemical Constituent Analysis of EAFVA with LC-HRMS

EAFVA phytochemical analysis using TSQ Exactive (Thermo) (LSIH, Universitas Brawijaya) in a gradient fashion at a flow rate of 40 L/min using Hypersil GOLD aQ 50 in a column 1 mm by 1.9 m; analysis time was 70 minutes. The flow rate for the mobile phase can be seen in Table 1. The compound finding from the analysis results was analyzed using the mzCloud software [15, 28].

### 2.11. Data Analysis

The data were presented as the mean value with a standard deviation (SD) and analyzed using SPSS v.22 software. All tests were repeated in triplicate.

## 3. Results

### 3.1. Minimum Inhibitory Concentration (MIC)

As shown in Table 2, EAFVA inhibited MRSA and *P. aeruginosa* with MIC values of 125 *μ*g/mL. Positive control tetracycline inhibited MRSA and *P. aeruginosa* with MIC values of 15.62 and 31.25 *μ*g/mL, respectively. This result indicated that EAFVA had a moderate antibacterial effect against selected clinical isolates (see Table 3).

### 3.2. Checkerboard Assay

**Figure 1:**
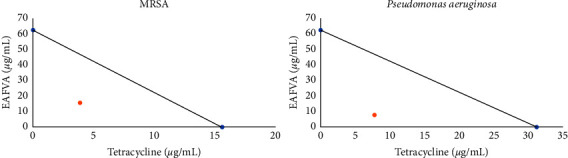
Isobologram of synergistic interaction between EAFVA and tetracycline against MRSA and *P.aeruginosa.*

The checkerboard assay was used to determine how EAFVA and tetracycline affected the tested bacteria. As presented in Table 4, the combination of EAFVA (15.62 *μ*g/mL) and tetracycline (3.9 *μ*g/mL) produced a 0.375 Fractional Inhibitory Concentration Index (FICI) for synergy against MRSA. On the other hand, the EAFVA enhanced the antibacterial activity of tetracycline against selected clinical isolates with a synergistic effect. It was found that EAFVA reduced the concentration of tetracycline, which suppressed the growth of MRSA. In contrast, the synergistic effect of the combination of EAFVA (7.8 *μ*g/mL) with tetracycline (7.8 *μ*g/mL) on *P. aeruginosa* was calculated to be 0.31. This showed that EAFVA could reduce the concentration of *P. aeruginosa* by 16 times (see Figure 1 for isobologram result).

### 3.3. Loss of 260 nm Absorbing Material

**Figure 2:**
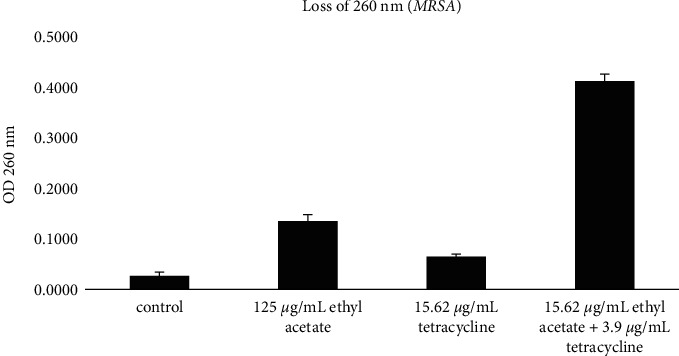
Loss of 260 nm absorbing materials on MRSA.

**Figure 3:**
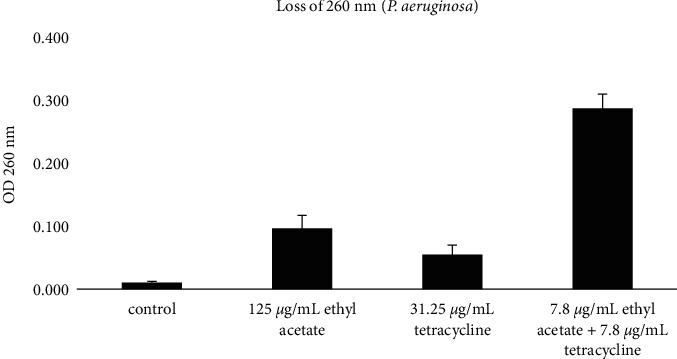
Loss of 260 nm absorbing materials on *P. aeruginosa.*

There was leakage of cells (nucleic acid components) in MRSA (Figure 2) and *P. aeruginosa* (Figure 3) from the EAFVA-treated supernatant. Leakage in cells treated with EAFVA and antibiotics alone showed absorbance at 260 nm in contrast to the untreated negative control without treatment. However, in the combination of EAFVA and tetracycline antibiotics, the leakage between nucleic acids or the release at 260 nm was increased in MRSA and *P. aeruginosa.*

### 3.4. Bacteriolysis Activity

**Figure 4:**
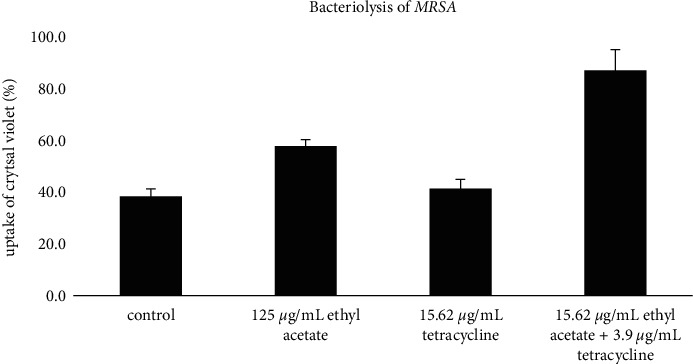
Bacteriolysis of the MRSA.

**Figure 5:**
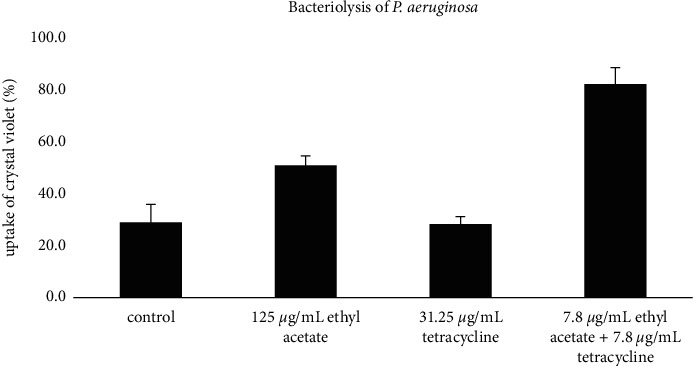
Bacteriolysis of the *P. aeruginosa.*

Based on the results of the bacteriolysis test, the percentage value of crystal violet absorption in treatment with tetracycline antibiotics with a concentration of 15.62 *μ*g/mL with a negative control showed an almost comparable value (Figures 4 and 5). However, compared to the negative control and single treatment with EAFVA plus tetracycline, the test with EAFVA treatment had a higher percentage of crystal violet absorption. Better results were shown in the combination of EAFVA with tetracycline antibiotics in both MRSA and *P. aeruginosa*, showing the same better results in the combination against these two test microorganisms.

### 3.5. Staphyloxanthin Assay

**Figure 6:**
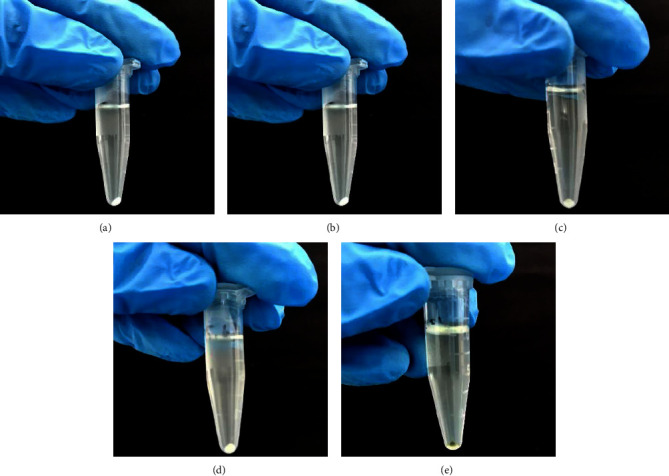
The golden-yellow color of staphyloxanthin production at (a) 125 *μ*g/mL concentration; (b) 62.5 *μ*g/mL concentration; (c) 31.25 *μ*g/mL concentration; (d) 15.625 *μ*g/mL concentration; and (e) negative control.

The staphyloxanthin test showed that its production could be seen and observed, which was marked by the formation of a golden-yellow color. With varying concentrations of samples treated with EAFVA, there is a decrease in the production of staphyloxanthin (Figure 6). In Figure 6(e), which is a microtube with a negative control cell, a golden-yellow color can be seen. A golden-yellow color was formed on the microtube at the lowest test concentration, 15.625 *μ*g/mL (Figure 6(d)). In comparison, the EAFVA test concentration is 125 *μ*g/mL (Figure 6(a)) and does not show the formation of a golden-yellow color on the microtube, likewise with the treatment at other concentrations. The staphyloxanthin test results revealed that its production might be seen inspected due to its golden-yellow color. In varying doses, staphyloxanthin production is diminished in the cell pellets isolated from EAFVA-treated samples (Figure 6). On the microtube of the negative control (Figure 6(e)), a golden-yellow hue can be noticed. At the lowest test concentration of 15.625 *μ*g/mL (Figure 6(d)), the formation of a golden-yellow color was observed in the microtubes. In contrast, the treatment with EAFVA at a concentration of 125 *μ*g/mL (Figure 6(a)) neither did not show the formation of a golden-yellow color in the microtubes nor in the treatment with other concentrations (see Figure 6).

### 3.6. Swarming Motility Assay

Based on the results of swarming motility, there were bacterial cells growing in the center of the Petri dish. In the medium containing EAFVA, there was little growth in the middle of the medium containing bacterial cells.

### 3.7. Phytochemical Constituent Analysis of EAFVA with LC-HRMS

The phytochemical analysis of EAFVA with LC-HRMS shows 15 constituents (Table 5).

## 4. Discussion

The minimum effective concentration is the amount of an antimicrobial compound that can stop bacterial growth under certain conditions and in a certain amount of time [29]. The results showed that at a concentration of 125 *μ*g/mL EAFVA, there was antibacterial activity that was significantly able to stop the growth of the two test bacteria (MRSA and *P. aeruginosa*). Based on its biological activity, EAFVA has phenolic molecules, especially in the flavonoid compound group. Due to this group of compounds, it is possible to have antibacterial activity. Flavonoids are a low-molecular-weight class of polyphenolic chemicals [30]. In another investigation, plant extracts containing rich flavonoids and pure flavonoids were tested to suppress the growth of pathogenic bacteria. Several mechanisms have been reported, such as those resulting from cell complexes with additional adhesion and developments in microbial inhibition [31]. EAFVA is highly effective against MRSA and *P. aeruginosa* [25].

Combining two types of antimicrobials has been described as one of the potential techniques to avoid the problem of antimicrobial resistance (AMR). The checkerboard assay was tested to evaluate the resulting interaction, namely, the synergistic interaction between the extract and commercial antibiotics [32]. In this test, the FICI was determined to prove the interaction between the EAFVA test sample and the tetracycline antibiotic. Thus, in combination, the MIC of EAFVA and Tetracycline decreased.

Apart from that, it shows a synergistic effect with no antagonism between EAFVA and tetracycline. Tetracycline works by attaching to the bacterial ribosome and engaging with the 30S subunit target of the 16S ribosomal binding. Tetracyclines impede translation by sterically interfering with the RNA transfer during elongation [33]. When used in conjunction with EAFVA, which was shown to contain flavonoid components such as luteolin, quercetin, apigenin, and other substances, it had a more significant impact than tetracycline alone. Tetracycline antibiotics and EAFVA work together synergistically, as evidenced by the increased effectiveness of combination therapy. In this mechanism, it is possible that EAFVA with Tetracycline has a different scenario where the site of action at the target is different. Secondary metabolites in EAFVA extract, such as flavonoids, possess a mechanism that can compromise the permeability of cell membranes. Whenever there is a disruption in the permeability of the cell membrane, it allows tetracycline to enter the cell and eventually occupy its working place. Previous studies reported a synergistic effect on nisin when mixed with an aldehyde from cinnamon. In contrast, nisin alone had modest antibacterial activity. When administered in combination, it provided better antibacterial activity, and the combination was considered successful [34].

In order to determine the mechanism of synergistic action between EAFVA and tetracycline, this study also focused on protein leakage and alteration of membrane cells. It was known that at a wavelength of 260 nm, the bacterial supernatant was measured, which was characterized by the absorption of absorbance and an increase in absorption; it indicated the occurrence of bacterial cell leakage or loss of nucleic acid material [35]. The combination between EAFVA and tetracycline was also studied. Compared with a single treatment, EAFVA and antibiotics with a combination of both showed an increase in absorbance at a wavelength of 260 nm. The result was confirmed using bacteriolysis activity. EAFVA and tetracycline altered MRSA and *P. aeruginosa* membrane cells by increasing crystal violet uptake. According to previous studies, it has been proven in previous reports that the treatment of microbiological infections with natural products combined with synthetic antibiotic mixtures can improve treatment and prevent microbiological resistance [36]. Combining *Eucalyptus camaldulensis* essential oil with polymyxin B antibiotics showed a synergistic effect on treatment-resistant *Acinetobacter burmanni* isolates [37]. Another study showed antiacne efficacy by combining two essential oils with tretinoin [38].

In addition, *α*-mangosteen and lawsone methyl ether also enhanced the antibacterial effect of ampicillin against several pathogens including MRSA by disrupting membrane cell permeability [39]. In another study, essential oils extracted from *C. maculatum* were used to inhibit the pathogens *Escherichia coli* DH5a and *P. aeruginosa* PAOI. The results of this study were similar to those obtained using the antibiotic colistin [40].

Another strategy to overcome antimicrobial resistance is with resistance restoration agents to detect antivirulence compounds. It allows the use of existing drugs and does not cause intense selection pressures that accelerate resistant colony growth [41]. As a virulence factor, *S. aureus*, specifically MRSA, can produce staphyloxanthin pigment [42]. The staphyloxanthin pigment distinguishes colonies of *S. aureus* from other staphylococci and Gram-positive bacteria. C30 gold keratinoid is a series of metabolic reactions embedded in a unique membrane in this pathogen known as staphyloxanthin [43]. Another study showed decreased staphyloxanthin pigment synthesis by L-ascorbyl 2,6-dipalmitate treatment with a more severe survival rate on whole blood analysis sensitivity testing on MRSA cells [44, 45].

Many types of bacteria in the laboratory show swarming, which is the movement of multiple cells with flagella on solid surfaces [46]. Biofilm production, colonization of plant interior and exterior surfaces, and pathogenicity or protective action on plant-associated bacteria can be significantly affected by the ability of organisms to swarm [47].

The antimicrobial effects of flavonoids are increasingly being recognized. In traditional medicine, crude plant extracts have been tested in vitro for antibacterial activity [48]. For example, flavonoids with antibacterial action have been isolated and their structures studied previously, for example, apigenin [49, 50], quercetin [51, 52], and others combined. The components of previous studies, such as apigenin, quercetin, luteolin, apigetrin, curom anine, and others, were in accordance with the results of the phytochemical constituent analysis of EAFVA with LC-HRMS in this study. At 78 *μ*g/mL, the luteolin component was associated with an antibacterial action against *Trueperella pyogenes* [53].

## 5. Conclusion

The antibacterial effect of EFVA with tetracycline on clinical isolates (MRSA and *P. aeruginosa*) revealed the antibacterial mechanism and activity with a synergistic effect.

## Figures and Tables

**Figure 7:**
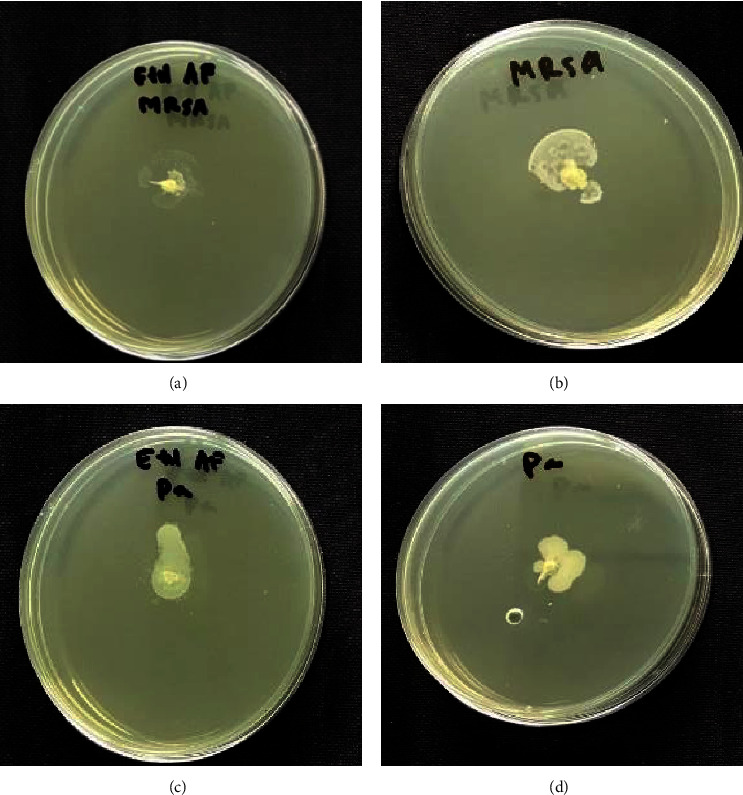
Swarming motility activity of EAFVA on (a) MRSA; (b) negative control MRSA; (c) *P.aeruginosa*; and (d) negative control *P. aeruginosa.*

**Table 1:** The ratio of flow rate and mobile phase.

No.	Time (min)	Mobile phase		Flow rate
		A (%)	B (%)	
1	0	Run		
2	0	96	4	40
3	40	80	20	
4	60	65	35	
5	61	5	95	
6	65	5	95	
7	65.1	95	5	
8	70	95	5	
9	70	Stop		

**Table 2:** Minimum inhibitory concentration of EAFVA against MRSA and *P. aeruginosa*.

Samples	MRSA (*μ*g/mL)	*P. aeruginosa* (*μ*g/mL)
EAFVA	125	125
Tetracycline	15.62	31.25

**Table 3:** MIC alone value and MIC combination value of EAFVA with the combination interaction on MRSA.

EAFVA-tetracycline	MIC a (*μ*g/mL)	MIC c (*μ*g/mL)	FIC	FICI	Interaction
EAFVA	125	15.62	0.125	0.375	Synergy
Tetracycline	15.62	3.9	0.25		

*Note.* a = alone; c = combination.

**Table 4:** MIC alone value and MIC combination value of EAFVA with the combination interaction on *P.aeruginosa*.

EAFVA-tetracycline	MIC a (*μ*g/mL)	MIC c (*μ*g/mL)	FIC	FICI	Interaction
EAFVA	125	7.8	0.06	0.31	Synergy
Tetracycline	31.25	7.8	0.25		

*Note.* a = alone; c = combination.

**Table 5:** Phytochemical constituent result of EAFVA with LC-HRMS.

No.	Name	Formula	Molecular weight	Retention time (min)
1	3-Methoxy-5,7,3′,4′-tetrahydroxy-flavone	C_16_H_12_O_7_	316.05678	0.834
2	Tectorigenin	C_16_H_12_O_6_	300.06191	0.843
3	4-Methoxycinnamic acid	C_10_H_10_O_3_	160.05173	5.216
4	5,7-Dihydroxy-2-(3-hydroxy-4[(2S,3R,4S,5S,6R)-3,4,5-trihydroxy-6-hydroxymethyl) oxan-2-yl]oxy}phenyl)4H-chromen-4-one	C_21_H_20_O_11_	448.09871	7.192
5	Quercetin	C_15_H_10_O_7_	302.04122	7.844
6	Apigetrin	C_21_H_20_O_10_	432.10396	7.918
7	Baicalin	C_21_H_18_O_11_	446.08296	7.999
8	Kuromanin	C_21_H_20_O_11_	448.09871	8.282
9	3-Methoxy-5,7,3′,4′-tetrahydroxy-flavone	C_16_H_12_O_7_	316.05678	8.786
10	Luteolin	C_15_H_10_O_6_	286.04613	9.602
11	Diosmetin	C_16_H_12_O_6_	300.06191	9.934
12	Apigenin	C_15_H_10_O_5_	270.05138	10.764
13	Ingenol-3-angelate	C_25_H_34_O_6_	430.23345	11.930
14	Chrysin	C_15_H_10_O_4_	254.05678	13.296
15	Glycitein	C_16_H_12_O_5_	284.06722	13.642

## Data Availability

Data sets used in this study are available from the corresponding author upon reasonable request.

## Abbreviations

MRSA: Methicillin-resistant* Staphylococcus aureus*
EAFVA: Ethyl acetate fraction of *Vernonia amygdalina* Delile leaves
PBS: Phosphate buffer saline
BHI: Brain heart infusion
MERO: Marine Education and Research Organization
MIC: Minimum inhibitory concentration
CFU/mL: Colony forming unit per milliliter
FICI: Fractional Inhibitory Concentration Index
FIC: Fractional inhibition concentration
OD: Optical density
LB: Lactose broth.
